# Moisture and material shape microbial communities in the built environment through disturbance–productivity relationships

**DOI:** 10.7717/peerj.21859

**Published:** 2026-11-02

**Authors:** Kaelyn Nannini, Dennis D. Krutkin, Scott T. Kelley, Adrian Ortiz-Velez, Jada D. Brown, Shawn Ogden, Fernanda Terrazas, Nicholas A. Barber

**Affiliations:** 1 Massachusetts Veterans Epidemiology Research and Information Center, VA Boston Healthcare System, Boston, MA, United States of America; 2 Biological and Medical Informatics Program, San Diego State University, San Diego, CA, United States of America; 3 Department of Biology, San Diego State University, San Diego, CA, United States of America; 4 School of Biological Sciences, University of California, San Diego, La Jolla, CA, United States of America; 5 Department of Obstetrics, Gynecology and Reproductive Sciences, University of California, San Diego, La Jolla, CA, United States of America

**Keywords:** Built Environment, Microbial diversity, Indoor microbiology, Microbiome, 16S ribosomal RNA, ITS, Fungal diversity, Network analysis

## Abstract

**Background:**

The built environment houses diverse microbial communities whose diversity and composition differ among building materials and environmental conditions. Ecological theory makes predictions about how productivity and diversity shape communities, and experiments in the built environment provide an opportunity to test these predictions. We manipulated moisture (constant or repeated wet–dry cycling) on three common building materials to test hypotheses that environmental disturbance interacts with the potential productivity of building materials to influence alpha and beta diversity.

**Methods:**

Coupons of different building materials, medium density fiberboard, gypsum wallboard and oriented strand board, were sterilized before passive inoculation, then kept wet or periodically air-dried before rewetting. Microbial communities were sampled using swabs every 14 days for 7-week period. Productivity was measured using bacterial cell counts, fungal hyphal length, and fungal spore density, and microbial diversity was determined via community amplicon sequencing using universal bacterial (16S) and fungal (ITS) primers.

**Results:**

The most productive material (oriented strand board) supported the highest bacterial alpha and beta diversity, and these diversity levels were reduced by repeated drying disturbances. Diversity patterns for fungi were more variable, with the highest alpha diversity on low–moderate productivity material (gypsum wallboard). Fungal beta diversity was reduced by disturbance on high productivity material but increased on the other materials. These patterns were driven largely by members of Bacillaceae, Sphingomonadaceae, and Aspergillaceae that reached high abundances in some treatments.

**Conclusions:**

Disturbance regime interacted with building material, so disturbance effects depended on material type. There were also differences between bacteria and fungi, which may be due to the scale-dependence of productivity–diversity relationships, but this pattern was not uniform. Network analysis found evidence of multi-species complexes of bacteria and fungi known to form biofilms, as well as evidence of competitive exclusion on specific materials and conditions. Together, these results indicate that disturbances can interact with building materials, and that these disturbances may be strongly mediated by biotic interactions.

## Introduction

The Built Environment (BE) is the diverse collection of human-made structures in the world, such as houses, restaurants, office buildings, and vehicles. Urbanization and industrialization have caused humans to shift towards an indoor environment, with one study showing Americans spend over 90% of their time indoors (Klepeis et al., 2001). These indoor environments—including both growth substrate materials and abiotic conditions—present novel selective pressures and habitats for microbial organisms, shaping the communities of microorganisms that humans encounter (Kelley & Gilbert, 2013; Gilbert & Stephens, 2018). Variation in community diversity and composition may have important consequences for microbial impacts in the BE, not only on building structure *via* degradation (Viitanen et al., 2010; Ling et al., 2014), but also to the overall health and well-being of occupants (Miller & McMullin, 2014; Dannemiller et al., 2016). Nonetheless, understanding of how BE microbial communities assemble is limited because BEs can be highly variable depending on a factors like material type, moisture, pH, temperature, geographic location, and surface location within the same BE (Viitanen et al., 2010; Kelley & Gilbert, 2013; Chase et al., 2016; Gilbert & Stephens, 2018). Moisture and high humidity in particular are key factors in inducing microbial growth (Du, Li & Yu, 2021), but moisture and humidity patterns vary widely among and within BEs.

Ecological theory provides a framework for predicting microbial community assembly in the BE (Costello et al., 2012; Miller, Svanbäck & Bohannan, 2018). Although developed primarily for macroecological communities, theoretical and empirical studies have identified both site productivity and disturbance regime as important community drivers (Grime, 1973; Kondoh, 2001; Haddad et al., 2008; Mouillot et al., 2013). Alpha diversity often exhibits a hump-shaped relationship along productivity gradients, particularly when measured at a local scale, so that the highest diversity occurs at moderate productivity (Chase & Leibold, 2002). This may be because diversity is resource-limited at low productivity and competition-limited at high productivity. However, frequent disturbances may disrupt this competition limitation, allowing more species to coexist in productive environments (Huston, 1999) (Fig. 1A). Alternatively, frequent disturbance could filter out disturbance-intolerant species, reducing diversity regardless of productivity. Productivity and disturbance also influence the multivariate composition of communities, again by acting as either potentially strong deterministic filters that limit community membership to particular taxa, or by reducing deterministic drivers and allowing stochastic processes to drive composition. The former of these outcomes may be more likely under low productivity, and the latter in high productivity environments, where other mechanisms, such as priority effects, could also lead to multiple compositional outcomes when disturbances are limited (Harrison et al., 2006; Chase, 2010). At the other extreme, the resource-limited conditions of low-productivity environments, and high disturbance frequency, are expected to homogenize communities (Chase, 2003; Jiang & Patel, 2008) (Fig. 1B).

**Figure 1:**
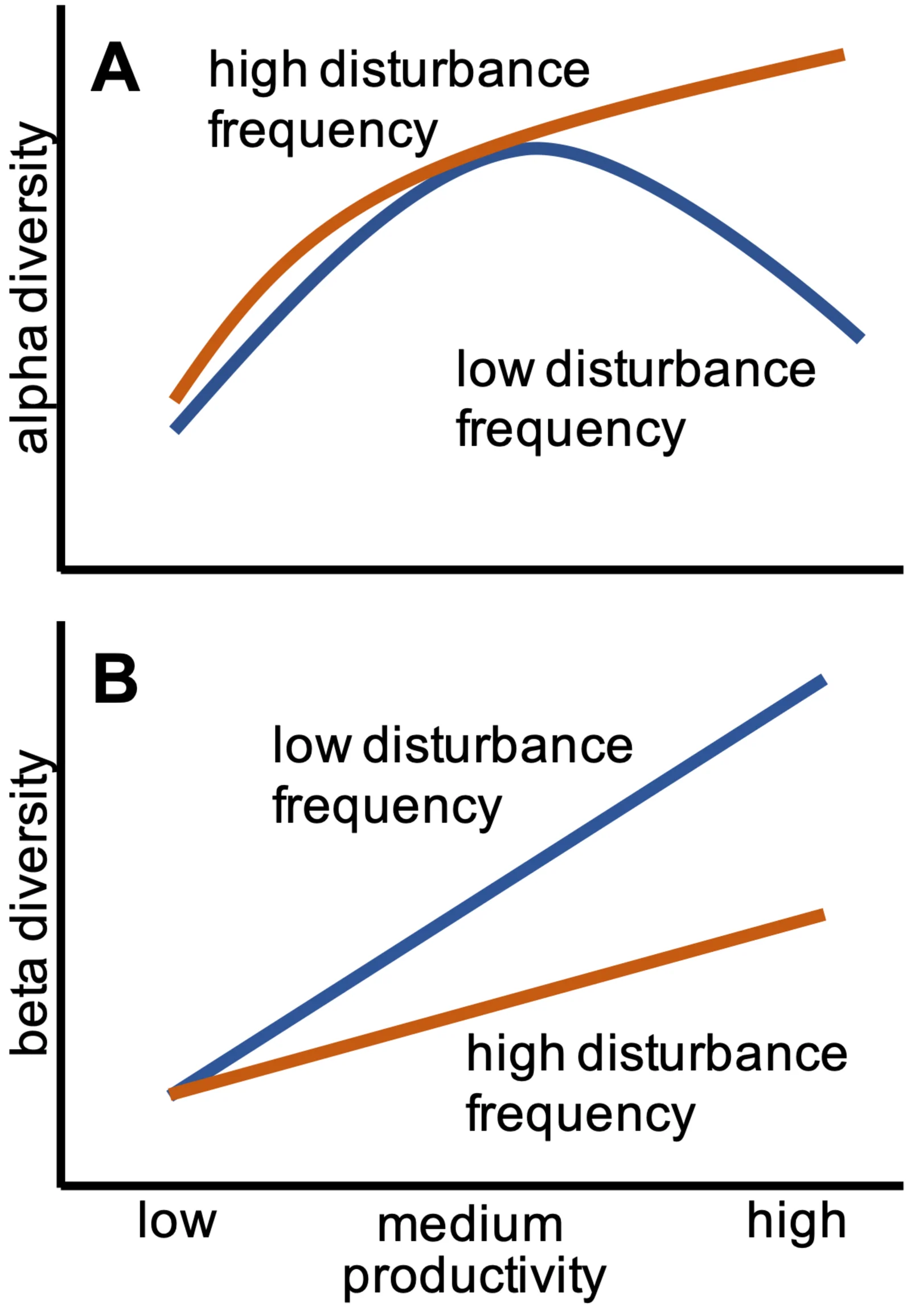
Theoretical patterns of disturbance on productivity. Hypothesized patterns of (A) alpha and (B) beta diversity *vs.* productivity under high and low frequency disturbance conditions.

In the BE, potential productivity differs among building materials that act as substrates for microorganisms, and drying acts as a disturbance that disrupts microbial communities. Some materials support copious fungal and bacterial growth, and others represent more resource-limited environments in which fewer microbes can establish and grow (Viitanen et al., 2010; Lax et al., 2019). The most important disturbance regimes in the BE are wetting/drying cycles that occur when building materials become moist from precipitation, plumbing problems, or other causes (Chase et al., 2016; Du, Li & Yu, 2021). Moisture allows microbes to proliferate following germination of spores and dormant cells (Pasanen et al., 2000), turning a dry “microbial wasteland” (Gibbons, 2016) into an active community, but subsequent drying is a disturbance that represents a stressful filter, limiting growth and potentially influencing diversity. Repeated wetting, followed by drying disturbances, creates a frequent disturbance regime that is likely to shape microbial community structure.

However, our ability to predict how conditions in our built environment shape communities of these microorganisms is limited (Adams et al., 2016), in part because much previous research often has focused on observational approaches. When building materials were experimentally maintained at either wet or dry conditions, bacterial and fungal diversity both declined over time, with bacterial diversity lower under consistent moisture (Lax et al., 2019). But this lower bacterial diversity was likely due to a subset of microbes increasing in abundance and reducing community evenness, compared to dry materials where activity was extremely limited, and the diverse community resulted from dormant microbes that accumulated with dust. A previous study that maintained moisture on two different materials, found differences in bacterial and fungal abundance between them (Xu et al., 2021). Notably, this study also demonstrated the importance of incorporating culture-independent counting methods for biomass estimates into community characterization, because some growth patterns were masked or appeared different when examining composition based on relative abundances alone.

In this study, we manipulated disturbance using a wet/dry cycling treatment (high disturbance) on three common building material types that differ in potential productivity and compared the resulting microbial communities to consistently wet (low disturbance) communities. We characterized bacterial and fungal communities and used quantitative profiling (combining biomass data and sequence data, *sensu* Vandeputte et al. (2017)) to examine effects on alpha and beta diversity. Our design and statistical analysis allowed us to test interactive effects of moisture manipulations and BE materials, which has not been done in similar previous studies. Based on predictions from community assembly theory (Fig. 1), we expected (1) that alpha diversity would increase with productivity under the disturbance treatment but would follow a hump-shaped pattern in low disturbance environments; and (2) that beta diversity, measured as mean pairwise dissimilarity within a treatment combination, would increase with productivity but be reduced by frequent disturbance.

## Methods

Portions of this manuscript were previously published as a preprint (Nannini et al., 2025).

### Experimental treatments and sampling

Three different building materials were used for this study: medium density fiberboard (MDF), gypsum wallboard (GW), and oriented strand board (OSB). These materials vary in their components and structure and thus represent contrasting environments for microbial growth (*i.e.,* potential productivity). Each material was cut into five cm x five cm “coupons”. Materials were UV sterilized, cleaned using 70% ethanol, and allowed to naturally inoculate for a period of 360 days (March 2020–March 2021) to represent a full annual seasonal cycle. Twenty coupons of each material type were positioned in open air on top of a shelf in a laboratory. There was minimal visitation to the laboratory during this time due to the COVID-19 pandemic. Although occupied spaces might include additional inputs from people, previous studies using similar methods with human occupancy did not find strong human bacterial or fungal signals (Xu et al., 2021). Following inoculation, coupons received one of two wetting treatments (*n* = 10 per treatment per material type). Each constant wet coupon was wetted with 5 g of UV-sterilized tap water and placed horizontally in separate sealed UV sterilized plastic tubs, representing a disturbance-free environment. Wet coupons were left wet and only disturbed for swabbing. Coupons undergoing the wet-dry cycling treatment, representing a repeated disturbance regime, were similarly wetted but after one week were drained and allowed to air dry in front of a HEPA filter approximately 24 h with the lid resting on top to prevent contamination of debris or particles, then resealed and incubated for a week before sampling and wetting again (Fig. 2).

**Figure 2:**
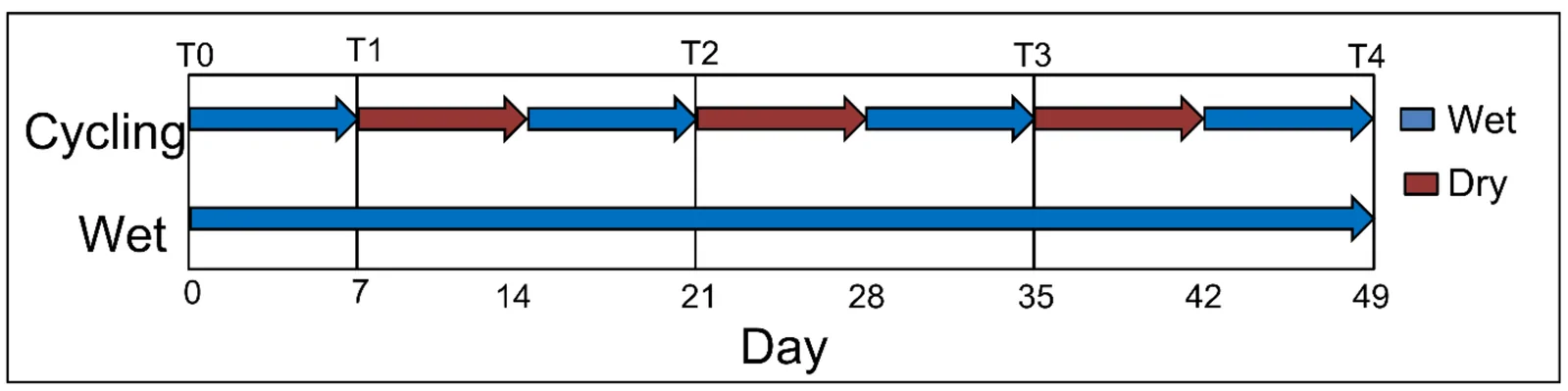
Sampling times for experimental treatments. Wet treatments maintained a consistently moist environment for microbial growth, while cycling treatments alternated through repeated drying period followed by rewetting before each sampling event.

Coupons were sampled at five timepoints: week 0 (start of the experiment), week 1, week 3, week 5, and week 7 (T0–T4 respectively), representing three cycles of drying disturbance for cycled coupons. At T0, two coupons of each material/treatment combination were sampled; for the subsequent time points, all coupons were sampled. In the Results (below), we focus on the endpoint (T4) because it represents the outcome of different disturbance regimes (*i.e.,* constant conditions *vs.* frequent, repeated drying disturbances), though we also show the patterns of bacterial and fungal growth, alpha and beta diversity, and select SV abundances over the different timepoints. We assumed the temperature and humidity conditions were consistent across the different environments. Samples were taken by opening the container, removing the coupon, and swabbing the surface of the coupon with a dry sterile polyester-tipped swab in one direction for approximately 20 s. The coupon was then replaced in the tub and sealed. The sample swab was broken off and placed in a 1.5 mL microcentrifuge tube containing 700 µL of 0.85% NaCl. The microcentrifuge tube was vortexed for 20 s. The sample was then aliquoted into separate microcentrifuge tubes: 200 uL for microscopy and 150 uL for DNA extraction. Aliquots for DNA analysis were stored on ice and extracted immediately after collection. Samples to be used for microscopy were fixed using a 4% formaldehyde solution and stored at 4 °C until further processing.

### Productivity

We used bacterial cell counts, fungal hyphal length, and fungal spore density to estimate microbial productivity. Epifluorescence microscopy was performed for approximately half of the samples at each sampling timepoint. Previously fixed samples were thawed, and 100 µL was resuspended in five mL of 0.02 µm filtered water. Samples were vacuum filtered, stained with 1X SYBR Gold, and allowed to incubate for 10 min in the dark before washing with 100 µL of filtered water for 5 min and mounting the filters onto glass slides. For bacterial biomass estimates, the slides were imaged under an Olympus 60X object magnification oil immersion microscope connected to a QImaging Retiga EXi Fast Cooled Mono 12-bit camera. Images were analyzed and counts were estimated using Image Pro software (Media Cybernetics, Rockville, MD, USA). For fungal counts, a Keyence BZ-X800E Series All-in-One Fluorescence Microscope was used for imaging. Spore counts were estimated using ilastik v1.4.0, and hyphae lengths measured in µM were analyzed using ImageJ v2.9.0 (Schneider, Rasband & Eliceiri, 2012).

### DNA extraction, sequencing, and processing

DNA extractions were performed for all samples using the QIAGEN DNeasy PowerSoil kit and stored at −80 °C until further processing. The extracted DNA was used to amplify target genes in the 16S rRNA gene and ITS region for bacteria and fungi respectively. For all samples, we targeted the V4 region of the bacterial 16S rRNA gene using the 515F and 806R primer pair (Walter et al., 2016). The highly variable fungal internal spacer (ITS) region 1 located between the 5.8S and 18S regions was amplified for half the samples using the ITS1 and ITS2 primer pairs (Walter et al., 2016). Reverse primers for both sets were barcoded to allow for re-identification of samples after sequencing. PCR conditions for both ITS and 16S amplification consisted of the following: (1) 94 °C for 3 min, (2) 94 °C for 45 s, (3) 50 °C for 1 min, (4) 72 °C for 1.5 min, (5) Cycling of steps 2–4, (6) 72 °C for 10 s, (7) 4 °C hold. Most samples underwent 30 cycles; however, a small subset of presumably low-biomass samples underwent 35 cycles due to amplification failure at 30 cycles. PCR was verified *via* gel electrophoresis on a 1% agarose gel. Sequencing was performed on the Illumina MiSeq platform by the Scripps Research Institute Genomic Center (San Diego, California USA).

Sequencing reads were imported into QIIME2 (11-2022 release) (Bolyen et al., 2019) for data processing. Samples were demultiplexed using the q2-cutadapt plugin. The data were denoised using the q2-dada2 plugin to generate sequence variants (SVs). The sequences were trimmed at 250 base pairs for 16S and 240 base pairs for ITS to remove positions with mean quality scores less than 30. To reduce the problem of excess zeros in the feature tables, SVs that were present in less than four and five samples in 16S and ITS, respectively, were removed from the analysis as determined by CurvCut v1.0 (Ortiz-Velez & Kelley, 2024). Sequences containing 16S SVs were taxonomically classified using the Silva database v138 (Quast et al., 2012) using the classify-sklearn option in the q2-feature-classifier plugin. For sequences containing ITS SVs, a Naïve-Bayes classifier was created using the UNITE fungal database v9.0 (Abarenkov et al., 2024) for taxonomic classification.

### Data analysis

Bacterial cell counts, fungal hyphae length, and fungal spore density were analyzed using linear mixed models with material type, disturbance treatment, sampling day, and their two- and three-way interactions as categorical fixed factors. Coupon ID was a random factor to account for repeated sampling. Both variables were log-transformed to meet assumptions of residual normality and homoscedasticity. Models were fit using the lmer() function in the lme4 package v1.1-38 (Bates et al., 2015) of R (R Core Team, 2022). Fixed factors were evaluated with type III *χ*^2^-tests using the Anova() function in the car package v3.1-5 (Fox & Weisberg, 2019).

We assessed alpha diversity using the Shannon diversity index, which reflects both richness and evenness. Although alpha diversity metrics are usually based on rarefied estimates to account for differences in read counts during sequencing, our read counts varied over several orders of magnitude because the material types we studied vary so widely in productivity (see results, below). Thus, rarefying to the level of samples with minimal read counts is impractical, and choosing an arbitrary minimal read count cutoff would bias sampling by disproportionately discarding certain treatment combinations (particularly in disturbed MDF samples, where microbial biomass was very low). Instead, we present unrarefied bacterial and fungal Shannon diversity values. Bacterial and fungal Shannon diversity were analyzed with linear mixed models using the same approach described above for productivity.

We examined beta diversity within treatments by calculating Aitchison’s distance on central log ratio (CLR) transformed read count data, with zeros replaced by a small value (0.01). CLR transformations and Aitchison’s distance are necessary because of the compositional nature of sequence data (Gloor et al., 2017). We focused on the final time point (T4) after repeated disturbance events had shaped community compositions and calculated pairwise values for all pairs of coupons within the same material x disturbance treatment combination. We analyzed these values using general linear models, with material type, disturbance treatment, and their interaction as fixed factors, using type III F-tests in the Anova() function. We also analyzed bacterial and fungal composition at the final sampling time using perMANOVA with the adonis2 function in vegan, including material, disturbance, and their interaction as predictor variables. We visualized final community composition using NMDS and mean abundance of identified family in material x disturbance treatments and present both relative and quantitative abundances. Quantitative abundances were estimates by multiplying relative abundances by bacterial cell counts and fungal spore density. To identify taxa driving differences among materials and treatments, we implemented random forest models using the randomForest package (v4.7.1.1) (Liaw & Wiener, 2002). For bacteria, two separate models were created, one for cycling coupons and another for wet coupons to compare the three material types because the combined dataset had a high error rate. Fungi were not separated by treatment. Features (SVs) with a Gini index value ≥1.0 were CLR-transformed and used as response variables in linear mixed models with the same parameters used in the biomass analyses.

### Network analysis

Bootstrapped analysis was performed using pybootnet v1.0 (Akhavan & Kelley, 2025) to test the robustness of correlation networks and to statistically compare network metrics among experimental treatments. Relative bacterial and fungal abundance counts were robust CLR (rCLR) transformed (Martino et al., 2019) with minimum value imputation prior to analysis, and 1,000 bootstrap replicates were run for every dataset. Spearman’s rank correlation networks were generated for each bootstrap network, and network statistics (number of edges, number of nodes, average degree centrality, transitivity, closeness centrality, betweenness centrality) were calculated for each network. Mean correlation coefficients (Spearman’s rho) were calculated from the bootstrap replicates for all pairwise correlations (bacteria-bacteria, fungi-fungi, and bacteria-fungi) and the final networks included correlations with mean rho ≥ —0.5—.

## Results

### Productivity

Productivity, as measured by bacterial counts, fungal hyphal length, and fungal spore density, differed among materials (Figs. 3A–3B, Fig. S1, Table 1). At the end of the experiment, productivity was greatest on OSB and was mostly increased by the wet treatment. Mean bacterial density was about four times greater on OSB than on MDF and GW for constant wet coupons, and six and four times greater, respectively, with the wet cycling disturbance. Disturbance reduced mean bacterial density by 64–77%. Mean fungal length of OSB was over five times greater than MDF and over eleven times greater than GW under the constant treatment. Fungal hyphae and spore patterns were similar but slightly weaker under the disturbed treatment, although MDF had limited fungal productivity with disturbance.

**Figure 3:**
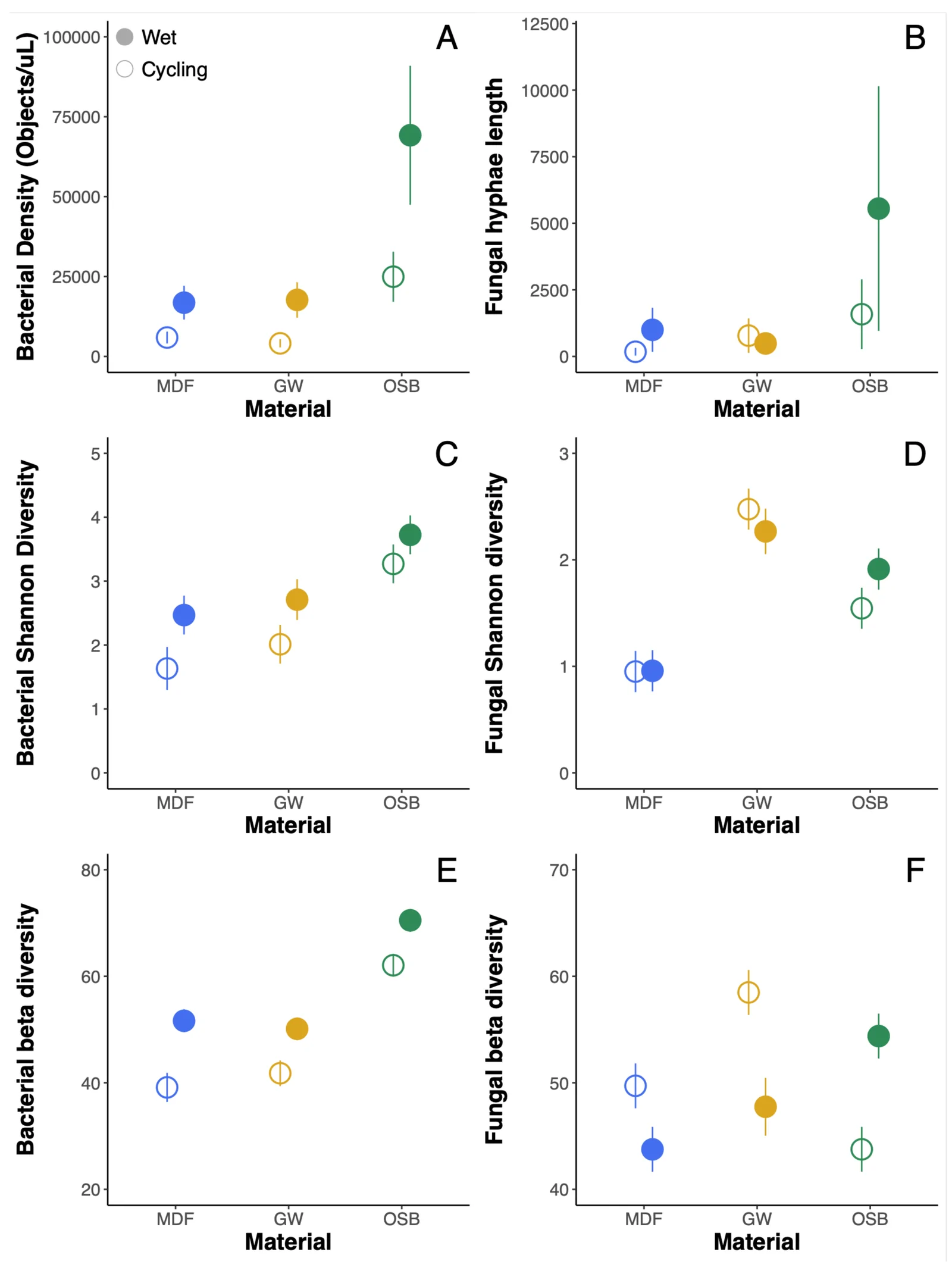
Relationship of biomass and ecological diversity to material type and disturbance. Productivity as measure by (A) bacterial counts and (B) fungal hyphae length on different building materials and under constant (solid symbols) or disturbed (open symbols) wet treatments. (C) Bacterial and (D) fungal Shannon diversity, and pairwise beta diversity (Aitchison’s distance) for (E) bacteria and (F) fungi. Values are estimated marginal means for the last sampling event (±1 SE).

**Table 1:** Results of linear mixed models analyzing effects of disturbance regime treatment and material type over time on productivity, alpha diversity, and beta diversity.

	Bacterial counts			Fungal hyphae length		
	*χ* ^2^	df	*P*	*χ* ^2^	df	*P*
Disturbance	42.38	1	<0.001	1.28	1	0.258
Day	78.11	3	<0.001	132.13	3	<0.001
Material	14.83	2	<0.001	44.22	2	<0.001
Disturbance × Day	11.03	3	0.012	1.16	3	0.763
Disturbance × Material	1.34	2	0.512	4.14	2	0.126
Day × Material	37.08	6	<0.001	30.07	6	<0.001
Disturbance × Material × Day	30.79	6	<0.001	9.59	6	0.143

	Bacterial Shannon			Fungal Shannon		
	*χ* ^2^	df	*P*	*χ* ^2^	df	*P*
Disturbance	6.25	1	0.012	0.39	1	0.535
Day	9.19	3	0.027	7.42	3	0.060
Material	15.82	2	<0.001	95.54	2	<0.001
Disturbance × Day	3.07	3	0.381	5.85	3	0.119
Disturbance × Material	3.01	2	0.222	0.07	2	0.966
Day × Material	39.15	6	<0.001	32.19	6	<0.001
Disturbance × Material × Day	14.06	6	0.029	14.72	6	0.023

	Bacterial Beta diversity			Fungal Beta diversity		
	*F*	df	*P*	*F*	df	*P*
Disturbance	26.96	1	<0.001	9.74	1	0.003
Material	57.04	2	<0.001	12.37	2	<0.001
Disturbance × Material	0.50	2	0.609	13.01	2	<0.001

### Alpha diversity

Bacterial and fungal alpha (Shannon) diversity differed over time among both disturbance regimes and materials, as indicated by a significant three-way interaction (Table 1, Figs. S2A–S2B). Bacterial diversity at the end of the experiment was greatest on OSB and reduced by the disturbance treatment (Fig. 3C). Thus, bacterial diversity increased with productivity under disturbance, as predicted. However, our hypothesis that high-productivity samples would have low alpha diversity in the absence of disturbance was not supported. Fungal alpha diversity showed different patterns, with the highest Shannon diversity on GW sampled with disturbance (Fig. 3D), and OSB diversity intermediate between the other two material types.

### Beta diversity

Beta diversity, as measured by mean pairwise Aitchison distance at the end of the experiment, was significantly affected by both material type and disturbance treatment (Table 1, Figs. S2C–S2D). For bacterial communities, beta diversity was greatest on OSB and reduced by the wet cycling disturbance treatment (Fig. 3E). However, there was no interaction between the material and cycling treatments as hypothesized, so the effect of cycling was similar among the three material types (Table 1). Fungal beta diversity patterns were different, with disturbance increasing beta diversity on both MDF and GW but reducing it on OSB (Fig. 3F). perMANOVA showed that taxonomic composition of bacterial communities was significantly shaped by material, disturbance, and their interaction, while fungal communities only differed significantly by material (Table 2, Fig. 4). Family composition of bacterial and fungal communities differed by material type and disturbance treatment (Fig. 5), with wet and cycling treatments different in composition most on GW, although there were also differences in the relative abundances of fungal families between treatments on OSB.

**Table 2:** Results of perMANOVA on bacterial and fungal community composition at the end of experiment by material type and disturbance treatment.

	Bacterial composition				Fungal composition			
	*F*	df	*P*	*R* ^2^	*F*	df	*P*	*R* ^2^
Disturbance	3.99	1	<0.001	0.05	1.13	1	0.210	0.04
Material	9.02	2	<0.001	0.23	1.57	2	0.001	0.11
Disturbance × Material	3.39	2	<0.001	0.09	0.89	2	0.815	0.06
Residual				0.64				0.79

**Figure 4:**
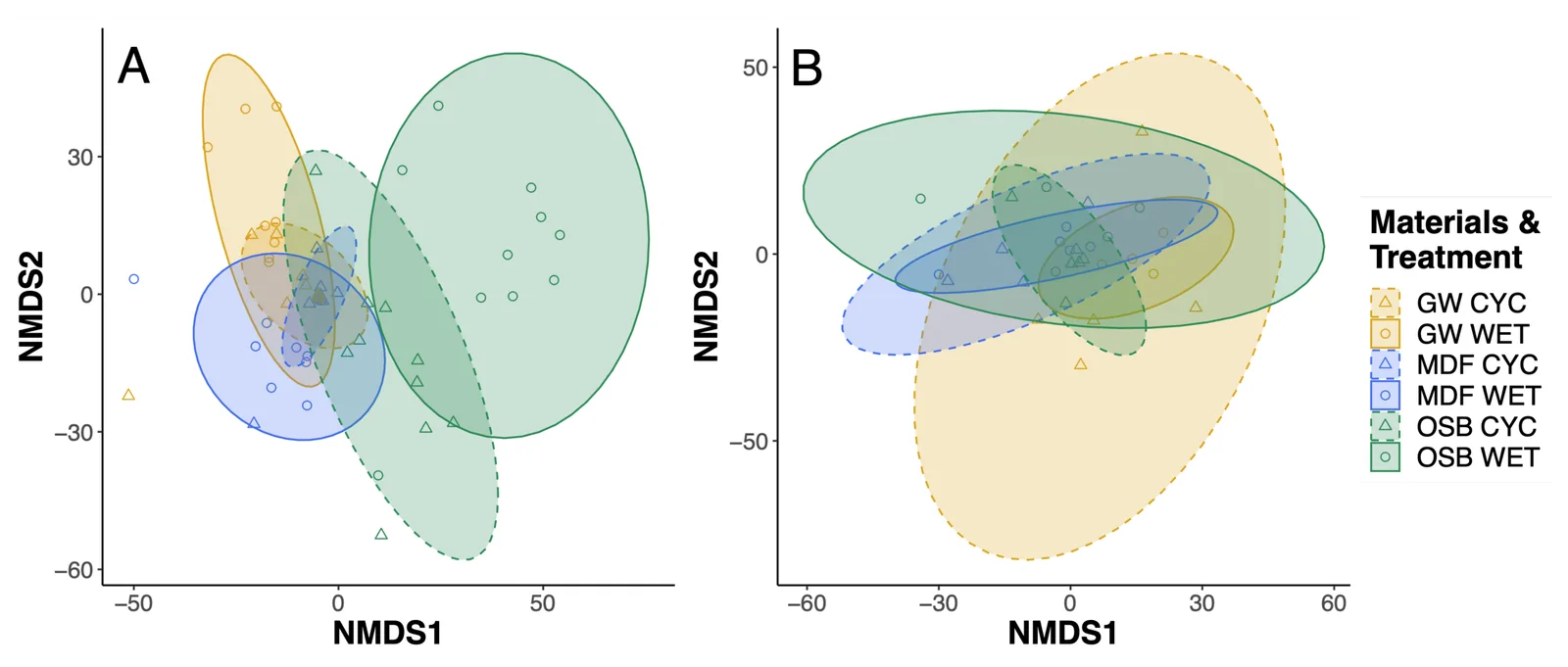
Visualization of beta diversity. NMDS plots of (A) bacterial and (B) fungal communities at end of experiment. Solid line ellipses and circles represent constant wet treatment, and dashed line and triangles represent cycling wet treatment.

**Figure 5:**
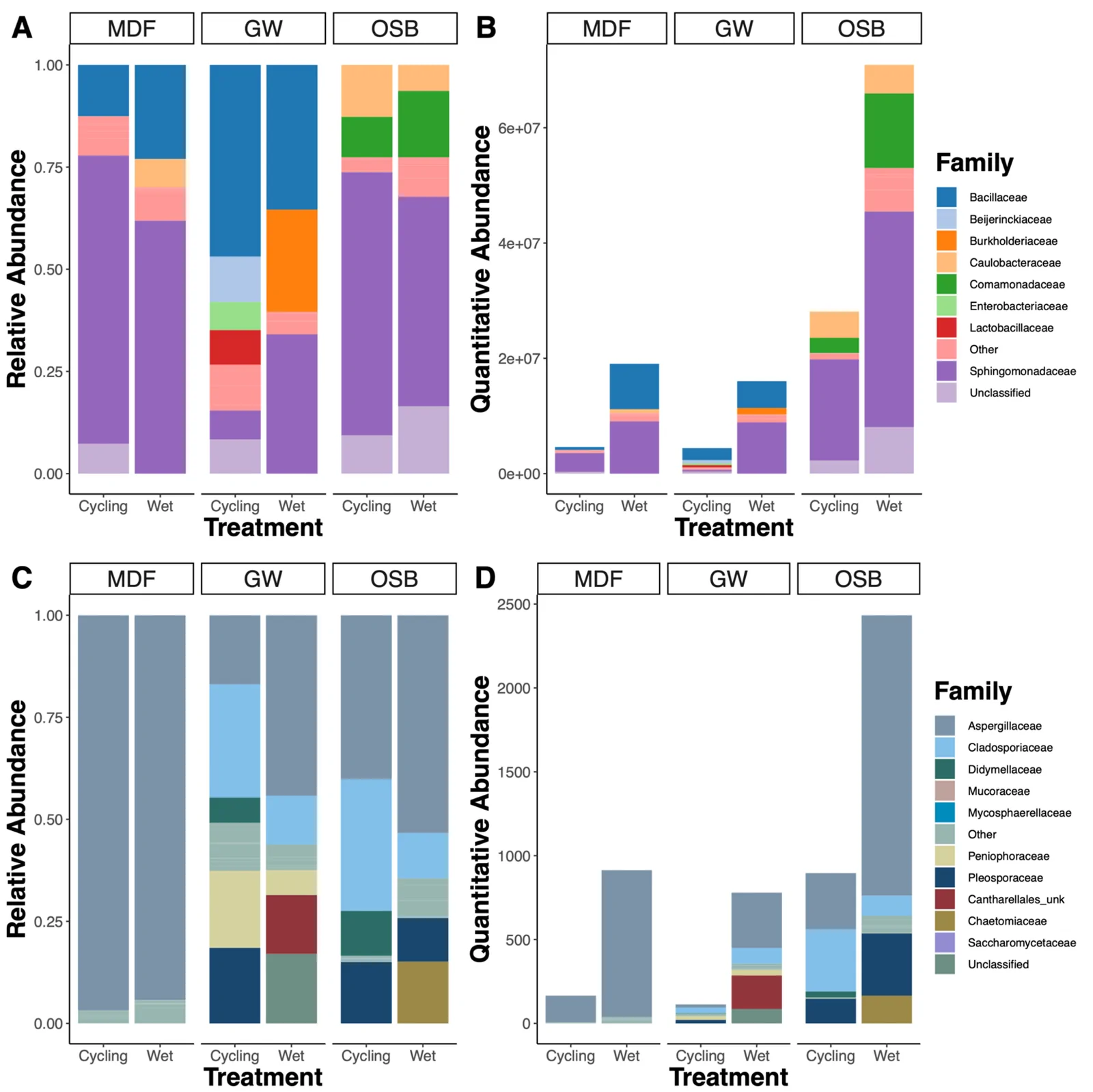
Relative and quantitative taxonomic abundances of bacteria and fungi on different materials. Family composition of (A, B) bacterial and (C, D) fungal communities on building materials by disturbance treatment at end of experiment.

### Key taxa

The two bacterial random forest models identified a combined 13 SVs with a Gini index over 1.0: nine *Bacillus* spp. and four *Sphingomonas* spp. Linear mixed effect models using each of these SVs as a response variable showed significant differences in abundance among material types (Disturbance x Material x Day interaction, *P* < 0.05 for all but two bacterial SVs), with *Bacillus* SVs consistently more abundant on GW and *Sphingomonas* SVs generally reaching higher abundances on OSB (Fig. 6). The fungal random forest model identified six SVs: three *Penicillium* spp., two *Cladosporium* spp., and one *Aspergillus* sp. Abundances of these SVs were significantly different among material types (all *P* < 0.02), with two *Penicillium* SVs (*P. brevicompactum* and *P. sumatraense*) and the *Aspergillus* SV generally more common on MDF. In contrast, two *Cladosporium tenuissimum* SVs were less abundant on MDF.

**Figure 6:**
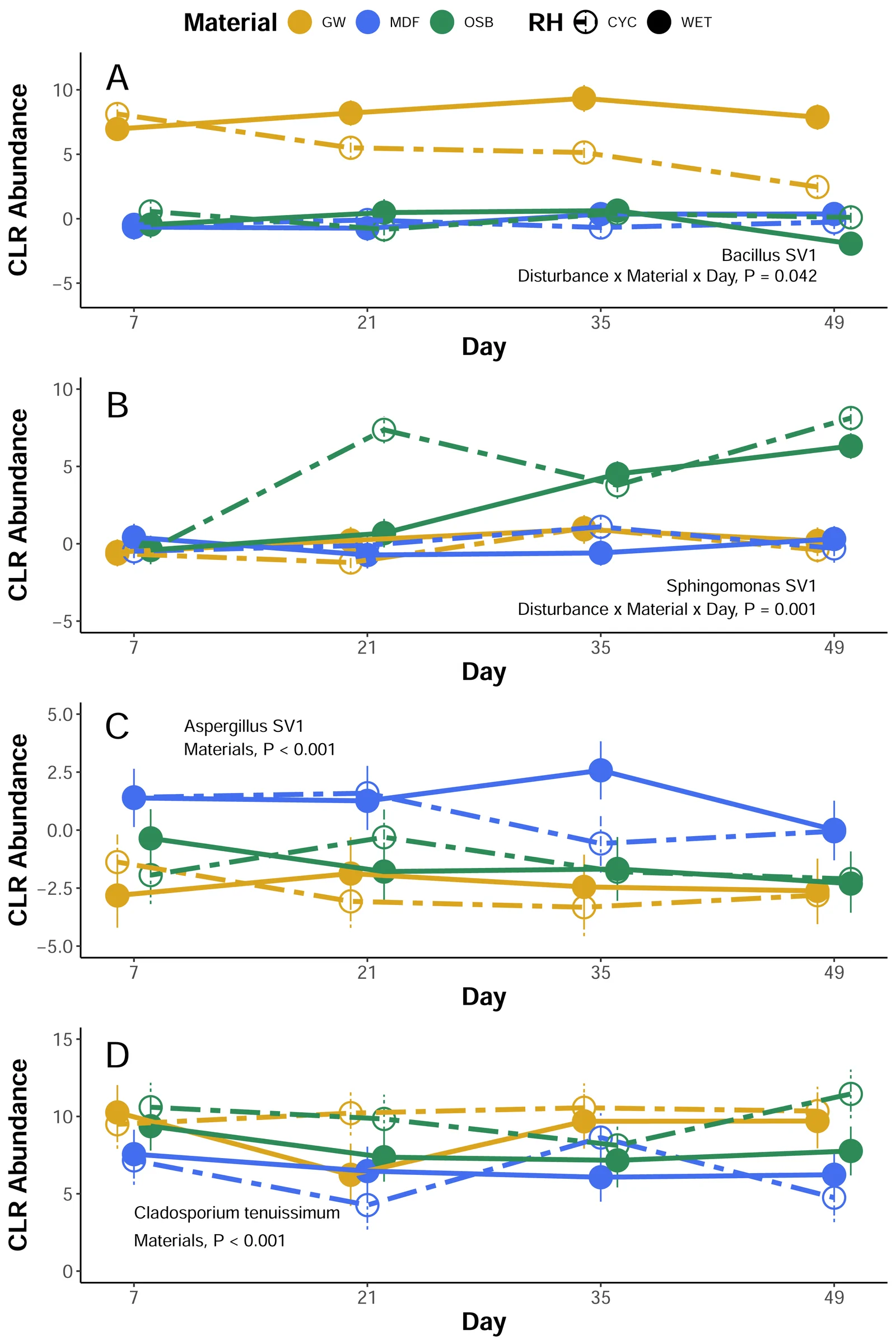
Relative abundance of four abundant bacterial sequence variants over time. Mean CLR abundances of individual *Bacillus* (A), *Sphingomonas* (B), *Aspergillus* (C) and *Cladosporium* (D) sequence variants identified as important *via* random forest classification over the course of the experiment. Dashed lines and solid lines indicate disturbed and wet treatments, respectively. Days 7, 21, 35, and 49 correspond to sampling times T1, T2, T3, and T4, respectively (see Methods).

### Network analysis

Bootstrap network analysis identified strongly supported suites of correlations within and between bacterial and fungal species on all the built environment materials. Significant differences were detected in between networks under different treatment conditions (wet *vs.* cycling) in the same materials and between different material types, with material type having the strongest effect (Fig. 7). The numbers of edges and nodes were higher in wet than cycling for MDF and OSB networks, but the reverse was true of the GW networks (Figs. 7A, 7B; 8; InteractiveNetworks.html). While the number of edges and nodes (bacteria or fungi) were similar between MDF and OSB, GW networks had fewer edges but many more nodes. Wet MDF and OSB coupons had higher number of negative correlations than their respective cycling conditions. GW networks had similar number of edges to the MDF and OSB networks, but GW had more nodes and lower density and degree centrality than the other materials. Most of the correlations were positive, but MDF and OSB networks included many negative correlations, with most of them occurring in the wet conditions between bacteria and fungi. GW included very few negative correlations overall in either condition (Figs. 7, 8; InteractiveNetworks.html).

**Figure 7:**
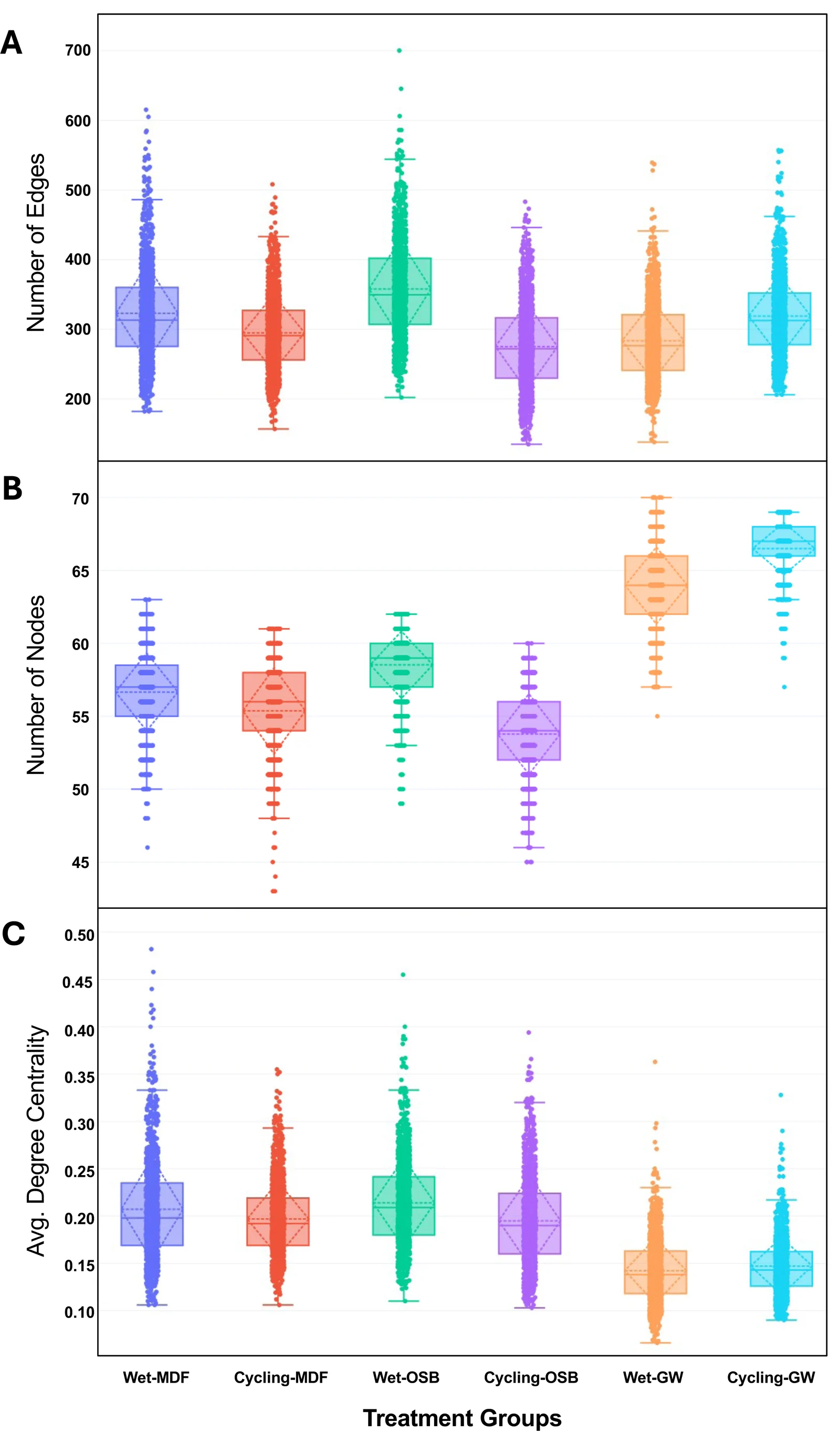
Comparisons of network metrics within and among materials. Box and whisker plots based on 1,000 bootstrap resampling of networks derived from samples from each treatment group comparing (A) number of edges, (B) number of nodes, and (C) average degree centrality. The edge values indicate total number of the pairwise correlations in each bootstrap replicate with rho > |0.5|. The number of notes indicated the total number of taxa in each replicate with at least one pairwise correlation to another taxon with rho > |0.5|. Degree centrality is the number of correlations of a given node, and average degree centrality is the mean degree centrality for an entire network, which was calculated for each bootstrap replicate. Higher average degree centrality indicates a more interconnected network. An interactive plot of the box and whisker plots for all the metrics can be found in the Supplemental Files.

## Discussion

Our experimental tests of ecological disturbance hypotheses with microbial communities on built environment building materials produced mixed results. As expected, the most productive material (OSB) supported the highest bacterial alpha and beta diversity, both of which were reduced by repeated drying disturbances. However, the patterns for fungi did not follow theoretical predictions (Fig. S3). The highest fungal alpha diversity occurred on the low to moderately productive material (GW). And while fungal beta diversity was reduced by disturbance on high productivity material, it increased on the other materials. Ecological theories of disturbance typically assume independent growth patterns among community members, but our network analysis found evidence of multi-species complexes of biofilm forming bacteria and fungi. This combined with evidence of competitive exclusion among biofilms suggests that disturbance was strongly mediated by biotic interactions.

**Figure 8:**
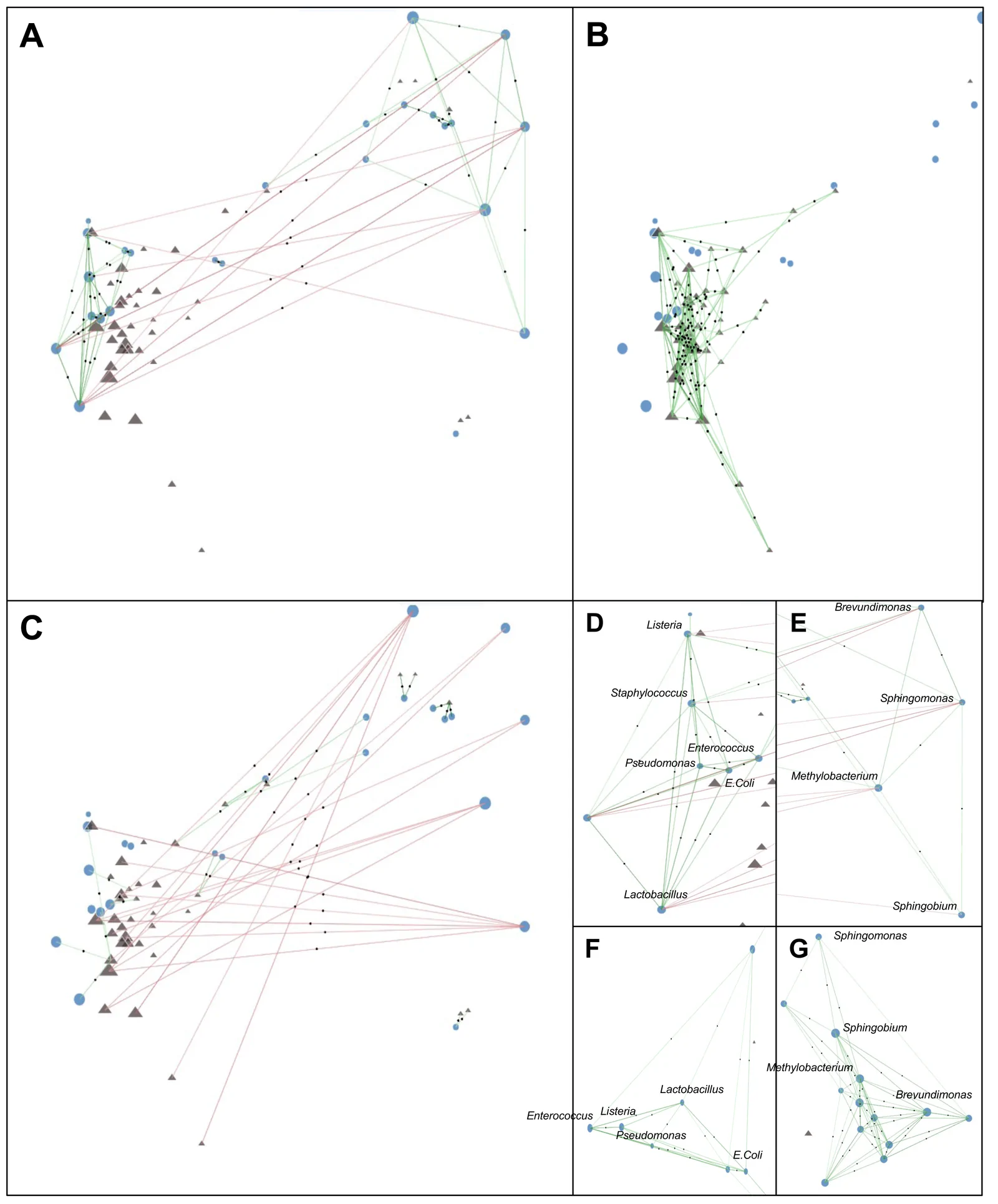
Visualization of network results. The circle nodes represent bacterial taxa, the triangle nodes are fungal taxa, and the lines (edges) connecting nodes indicate correlations with mean rho > |0.5| from 1,000 bootstrap replicates. Positive correlations are in green and negative correlations in red. Node size indicates the number of correlations of a particular taxon (degree centrality) with the lager nodes having more total correlations with other nodes. These networks include MDF-Wet (A) bacteria-bacteria, (B) fungal-fungal and (C) bacteria-fungal correlations. Graphs (D) and (E) show closeup of two bacteria-bacteria sub-graphs with in the MDF-Wet network, while (F) and (G) represent two subgraphs of the OSB-Wet network. An interactive plot of all the constructed networks can be found in the Supplemental Files.

Bacterial and fungal growth were both significantly higher on one building material, OSB, indicating that it provides high potential productivity as a substrate for microbial communities (Figs. 3A–3B). Although we predicted that alpha diversity would follow a unimodal relationship in the absence of disturbance and increase under repeated disturbances, bacterial and fungal diversity patterns differed. Bacterial alpha diversity responded similarly to disturbances at all productivity levels, maintaining high diversity on OSB, while fungal alpha diversity was highest on GW, a low- to moderate-productivity substrate. Our expectation that a positive relationship between beta diversity and productivity would be strongest under low disturbance was partially supported: bacterial beta diversity was greatest on OSB, but beta diversity was similarly reduced by disturbance on all materials. Fungal beta diversity was increased by disturbance on low-productivity materials but reduced on OSB. Below, we discuss possible explanations for these patterns, which microbial taxa may have driven the results, and the implications for understanding and predicting microbial communities the BE.

### Alpha and beta diversity

A unimodal relationship between productivity and diversity is often assumed because more species are supported along increasing resource gradients, but at the highest productivity levels, a competitively dominant subset of the community can limit diversity (Huston, 1999). We expected that disturbances, in the form of repeated drying, would limit this competitive exclusion, permitting higher diversity on the most productive material. However, this did not occur, and material–disturbance interactions on Shannon diversity were weak, especially for bacteria (Figs. 3C–3D). Disturbance generally reduced alpha diversity, including for both bacteria and fungi on OSB, the most productive material. One possible explanation for these alpha diversity patterns is the scale-dependence of productivity–diversity relationships. Unimodal patterns may emerge when assessed at local scales, while at larger scales diversity increases consistently with productivity. Chase & Leibold (2002) found a hump-shaped relationship when sampling individual ponds within watersheds, but a linear relationship when comparing among entire watersheds. Similarly, especially given the size differences between single-celled bacteria and much larger multicellular fungi, we may have been sampling bacteria at a regional scale (essentially pooling across many local environments with each swab across a coupon), whereas fungal samples better represented the local coupon scale at which interspecific interactions occur. Characterizing bacterial communities at the local scale may require much more localized sampling within coupons, although it may be challenging to sequence such low-biomass samples. An additional caveat is that, even if unimodal productivity–diversity relationships exist, it can be difficult for investigators to know where along the productivity gradient the studied environments lie. That is, OSB was clearly the most productive of these three materials, but it could still be quite low relative to other growth surfaces containing more (or more diverse) substrates in the BE or elsewhere (Gibbons, 2016).

We also expected variation in community composition among coupons, or beta diversity, to depend on material type and disturbance treatment. Both low productivity and frequent disturbance can function as environmental filters, limiting community membership to species with tolerance for limited resources or resilience against disturbances (Chase, 2003; Jiang & Patel, 2008). These processes would result in a consistent, limited set of species persisting in these environments and low among-community beta diversity. The prediction that beta diversity would increase with productivity was partially supported: bacterial beta diversity was highest on high-productivity OSB regardless of disturbance, and fungal beta diversity was highest on OSB when disturbance was absent (Figs. 3E–3F). These effects are visible in the wide dispersion of OSB samples in ordinations (Figs. 4A–4B). However, under frequent disturbances, fungi on OSB had surprisingly limited compositional dispersion (Figs. 3F & 4B), indicating that a limited number of taxa may have been able to take advantage of the resource availability following disturbances and reach high abundances on these coupons. The identity of these taxa could have important practical implications if they tend to be species that cause physical degradation of their substrate or cause health problems.

### Taxonomic composition

Understanding which taxa drove the diversity patterns discussed above may help further reveal the ecological mechanisms shaping these microbial communities. Importantly, we show how combining relative abundances of bacteria and fungi with counts of cell (bacteria) or spore (fungi) density to generate quantitative abundances, gives a clearer picture of community differences (Xu et al., 2021).

Within bacterial communities, several *Sphingomonas* SVs were typical of OSB in general, and members of Sphingomonadaceae more broadly comprised much of the bacterial communities, except on GW subject to the drying disturbance. If *Sphingomonas* are particularly susceptible to drying, then the thin surface paper of GW may have limited their persistence in comparison to wet samples or the other two wood-based materials that may maintain moisture longer. Bacillaceae were also significant components of bacterial communities, except on OSB. Even under drying disturbances, they persisted on MDF and GW coupons, albeit at low biomass. Several *Bacillus* SVs were also identified as distinguishing GW from the other materials. In a similar experiment, Lax et al. (2019) found that *Bacillus* reached high relative abundances on consistently wet GW but remained rare on other materials or when coupons were kept dry. The persistence of *Bacillaceae* on wet-cycling GW and MDF coupons in our experiment suggests that they may be tolerant of low-productivity and high-disturbance conditions, possible due to their well-known ability to form spores.

In fungal communities, two *Penicillium* SVs and an *Aspergillus* SV were identified as characteristic of MDF coupons. These and other Aspergillaceae were the dominant components of MDF communities, although they were a large part of GW and OSB communities, reaching especially high abundances in wet conditions. These findings align with other studies of the BE, in which Aspergillaceae is often common or dominant. On MDF and GW, the greater relative and quantitative abundance of Aspergillaceae on wet coupons was associated with reduced beta diversity among these communities, whereas the higher quantitative abundance due to wet treatment on OSB increased beta diversity. One explanation is that the same SVs reached high abundances on the low-productivity MDF and GW coupons, homogenizing these communities, while on OSB different taxa might have become common and driven the high inter-site compositional variation. If mechanisms like priority effects or ecological drift (Chase, 2007; Fukami, 2015) are more important under higher productivity, that could have driven the opposite disturbance effects on OSB. This pattern might be widespread on other building materials that, like OSB, are prone to copious microbial growth.

One important caveat to these interpretations is that the idea of environmental filtering as a structuring force in communities may overlook biotic interactions (Kraft et al., 2015). Previous network analysis of microbes on building materials by Lax et al. (2019) found potential interactions between bacteria and fungi possibly mediated by antibiotics. Our bootstrap network analysis of communities on different materials and conditions also strongly suggested a role for biotic interactions in shaping differential community responses. For example, we observed higher levels of connectivity in wet MDF and OSB respective to cycling, with the reverse pattern true with GW (Figs. 7A, 7B). The higher degree centrality and density of the MDF and OSB indicated a higher level of interactions among organisms in these conditions compared with the GW, which had many correlations (edges), but each taxon node was not as highly connected (Fig. 7C). Only a few nodes in the GW networks had more than 10 degrees (10 or more edges), while there were many highly connected nodes in the MDF and OSB networks (InteractiveNetworks.html). The number of negative correlations also differed markedly among conditions (Fig. 8; InteractiveNetworks.html): wet MDF and OSB coupons had higher number of negative correlations than their respective cycling conditions and only one negative correlation was detected in at all in the OSB cycling networks. This suggests a much higher level of competition in the wet conditions than cycling, an idea supported by the consistently higher levels of growth on all wet materials (Fig. 5). Most, and sometimes all, of the negative correlations occurred between fungi and bacteria which are well known to strongly compete for resources in soils (Wang & Kuzyakov, 2024). While MDF did not have the same amount of overall cellular growth as OSB, MDF coupons had more negative correlations (fungi-bacteria and bacteria-bacteria; Fig. 8). On the other hand, GW networks had very few negative correlations, suggesting that competition may not play much of a role in structuring the GW microbiome in the relatively simple, low-resource environment of this material.

Visualizations of the bootstrapped networks identified several interesting clusters (subgraphs) of organisms appearing in more than one material. The most consistent subgraph detected in every network material was the *E. coli-Enterococcus-Lactobacillus-Listeria-Pseudomonas-Staphylococcus* cluster (Figs. 8D, 8F; InteractiveNetworks.html). These bacteria include many potential pathogens that can be multidrug (antibiotic) resistant. The level of taxonomic resolution afforded by 16S cannot confirm the pathogenicity of any of the microbes growing on these materials, but this subgraph strongly resembles the multi-species *Pseudomonas aeruginosa* biofilms found in hospital medical devices (Cendra & Torrents, 2021). *P. aeruginosa* exudes a polysaccharide- and environmental DNA (eDNA)- based extracellular matrix that supports the growth bacteria including *Staphylococcus aureus* and *Enterococcus faecalis*, as well as fungi and even viruses (Cendra & Torrents, 2021). This suggests that similar types of biofilms could form on many given indoor surfaces. Interestingly, *Pseudomonas* was not the most highly connected node in these subgraphs and the multispecies composition differed from the biofilms found in medical devices.

We also observed a subgraph of positively correlated fungi appearing on all the materials that included *Aspergillus*, *Penicillium*, *Didymellaceae*, and *Chaetomiaceae*. The biofilm formation of *Aspergillus fumigatus* has been extensively studied, and it is also composed of polysaccharides and eDNA among other polymers and can also exist as a multispecies complex, though little is known about the interspecific interactions (Wang et al., 2024).

The final common subgraph network we identified was the *Brevundimonas-Methylobacterium-Sphingobium*-*Sphingomonas* cluster, which was most pronounced on wet MDF and OSB and not identifiable on GW. *Sphingomonas, Sphingobium,* and *Methylobacterium* all make biofilms and are commonly found together in nature along with other organisms, especially on leaf surfaces (Schlechter & Remus-Emsermann, 2025). Species within these genera co-aggregate together and with other species in very wet BE habitats, such as drinking water systems and shower curtains (Kelley et al., 2004). *Brevundimonas* also forms biofilms and is commonly found in wet BE habitats such as showerheads and, intriguingly, is a problematic slime depositor in paper mill machinery (Rättö, Suihko & Siika-aho, 2005) that processes wood and paper, the primary components in our materials.

While the species in the *Sphingomonas* cluster were positively correlated with one another they were negative correlated with many fungi and a few bacteria. The number of negative correlations were especially pronounced on MDF-Wet conditions (Figs. 8A, 8C) suggesting a degree of competitive exclusion on certain materials. The combination of mutualistic multispecies biofilms on the one hand and competitive interactions among different biofilms on the other could significantly impact patterns of alpha and beta diversity (Maynard et al., 2017; Tu et al., 2019). For instance, a high level of cooperation and/or competition could explain the lower level of beta diversity in the wet MDF and OSB compared to the GW (Fig. 3). These types of environment-specific dynamics may help explain why patterns of alpha and beta diversity varied by environment and taxonomic group. Future work should investigate the importance of biofilm mutualisms and antagonism in shaping the biodiversity of BE materials.

### Implications

Microbial growth in the BE can represent an economic, safety, and health threat because of the potential for material degradation, toxin production, and pathogen spread (Viitanen et al., 2010; Miller & McMullin, 2014; Gilbert & Stephens, 2018). Shifts in the diversity and composition of microbial communities could promote or suppress the agents of these negative impacts, but the community-level interactions that determine this are poorly known. Changing precipitation patterns under climate change may alter wetting frequencies or patterns, driving the sorts of community variations we document in this study. For example, consistent moist conditions, which could arise in areas where rainy seasons last longer or rainfall events become more frequent, promote many *Aspergillus* spp., including those that produce toxins that threaten human health (Prenafeta-Boldú, Medina-Armijo & Isola, 2022). Further, we have shown here that different wetting regimes may interact with BE materials to constrain (reduced beta diversity) or amplify (increased beta diversity) compositional variation. This was evident in fungal communities on GW, which had higher beta diversity under the wet cycling treatment. High beta diversity, or wide variation in community structure among samples experiencing the same conditions, means that it may be very difficult to predict important aspects of community composition—such as the presence of potentially threatening microbes—under the sort of wetting and drying cycles that can occur with roof leaks and periodic precipitation.

Microbial-resistant building materials, such as “mold-free” GW or material coatings that limit bacterial and fungal growth, hold promise for limiting potential BE degradation or health effects (Wu & Wong, 2020). But the same issue of low predictability of which particular taxa succeed on these materials applies: although we found that lower productivity of bacterial communities was associated with lower alpha and beta diversity (*i.e.,* more constrained and consistent combinations of bacteria taxa), the high beta diversity of fungal communities on low-growth GW means that even when fungal growth is limited, the species that are able to succeed may be vary from one sample to another, even if they were initially inoculated in the same location. Studies targeting the occurrence of particularly harmful fungi (or bacteria) may help reveal if there are specific conditions, including broader community context, that require careful attention to reduce threats in homes, schools, workplaces, and other parts of the BE.

## Supplemental Information

10.7717/peerj.21859/supp-1 Supplemental Information 1 Microscopy count results for bacteria and fungi (A) Bacterial density, (B) fungal hyphae length, and (C) fungal spore density by material and disturbance treatment over the course of the experiment. Dashed lines and solid lines indicate disturbed and wet treatments, respectively. Days 7, 21, 35, and 49 correspond to sampling times T1, T2, T3, and T4, respectively (see Methods).

10.7717/peerj.21859/supp-2 Supplemental Information 2 Alpha and beta diversity on building materials over time Mean Shannon (alpha) diversity for bacterial (A) and fungal communities (B); Mean beta diversity for bacterial (C) and fungal diversity (D) disturbed wet treatments over the course of the experiment. Dashed lines and solid lines indicate disturbed and wet treatments, respectively. Days 7, 21, 35, and 49 correspond to sampling times T1, T2, T3, and T4, respectively (see Methods).

10.7717/peerj.21859/supp-3 Supplemental Information 3 Comparisons of theoretical to observed patterns of disturbance on productivity Bacterial (C, D) and fungal (E, F) diversity compared to measures of productivity. Hypothesized relationships from Fig. 1 are reproduced (A, B) for comparison. Values are estimated marginal means ± 1 s.e. Open symbols are the cycling treatment, which corresponds to the orange line in A and B (high disturbance frequently), and closed symbols are the constant wet treatment (blue line, low disturbance frequency).

10.7717/peerj.21859/supp-4 Supplemental Information 4 Metadata for data analysis in a text csv format raw data counts for microbial cells, fungal spores, fungi hyphal lengths and ATP measurments.

## Additional Information and Declarations

### Data Availability

The following information was supplied regarding data availability: The data is available in the Supplemental Files. The data and code are available at Zenodo: Kelley, S., Barber, N., Kaelyn, N., Krutkin, D., Ortiz-Velez, A., & Fernanda, T. (2026). Moisture and material shape microbial communities in the built environment through disturbance–productivity relationships (Computer software). Zenodo. https://doi.org/10.5281/zenodo.20723866
The raw sequence data are available at NCBI BioProject: PRJNA1445366, PRJNA1445489.
