# Supplementary material for: Moisture and material shape microbial communities in the built environment through disturbance–productivity relationships
Source: PeerJ. 2026 Nov 2;14:e21859. doi: 10.7717/peerj.21859 (PMC13738189; doi:10.7717/peerj.21859)

Figure S1: Microscopy count results for bacteria and fungi.
(A) Bacterial density, (B) fungal hyphae length, and (C) fungal spore density by material and disturbance treatment over the course of the experiment. Dashed lines and solid lines indicate disturbed and wet treatments, respectively. Days 7, 21, 35, and 49 correspond to sampling times T1, T2, T3, and T4, respectively (see Methods).

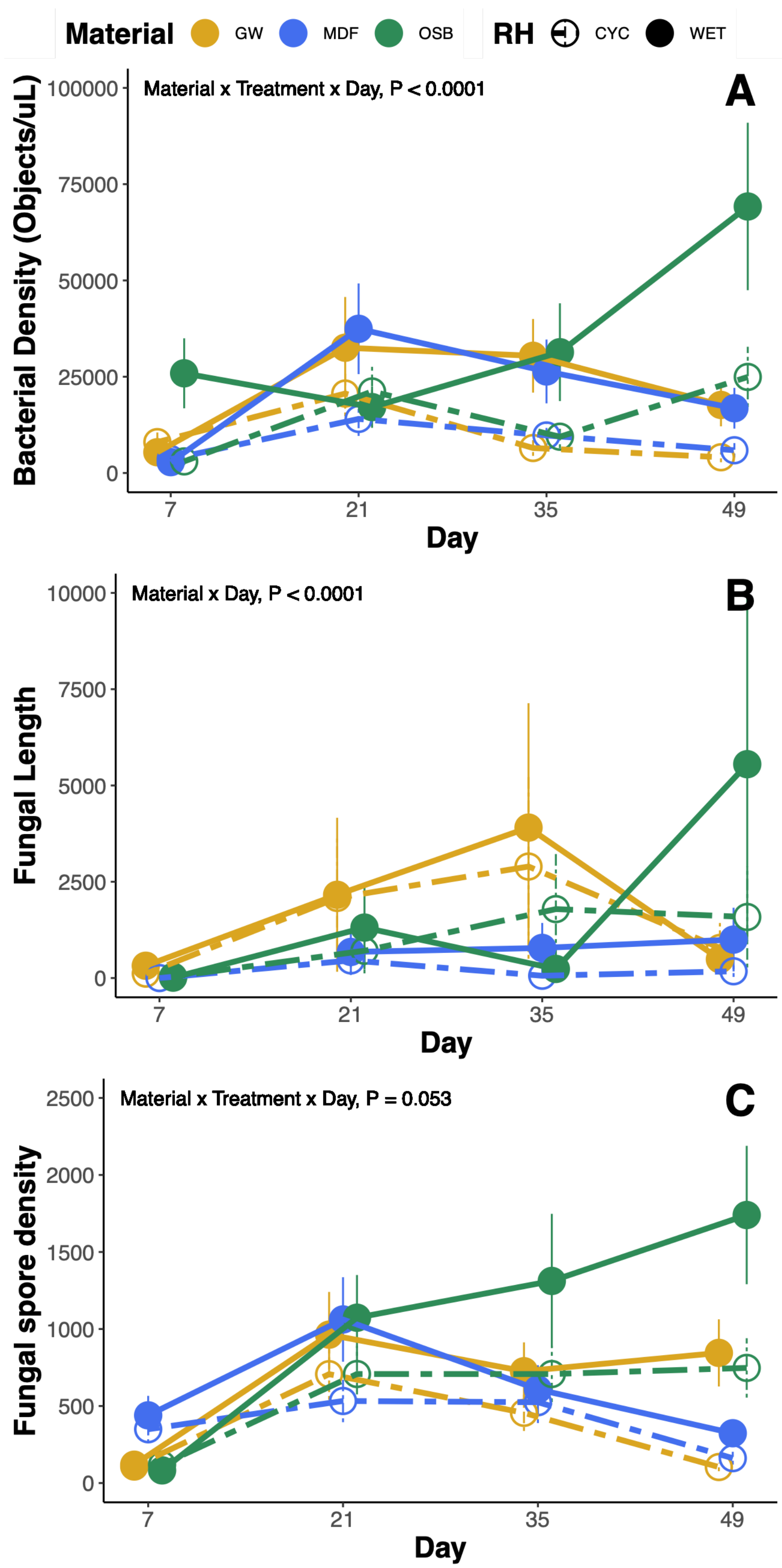

Supplement: Supplementary file 1 [file peerj-14-21859-s001.pdf]
