# Supplementary material for: Moisture and material shape microbial communities in the built environment through disturbance–productivity relationships
Source: PeerJ. 2026 Nov 2;14:e21859. doi: 10.7717/peerj.21859 (PMC13738189; doi:10.7717/peerj.21859)

Figure S2: Alpha and beta diversity on building materials over time.
Mean Shannon (alpha) diversity for bacterial (A) and fungal communities (B); Mean beta diversity for bacterial (C) and fungal diversity (D) disturbed wet treatments over the course of the experiment. Dashed lines and solid lines indicate disturbed and wet treatments, respectively. Days 7, 21, 35, and 49 correspond to sampling times T1, T2, T3, and T4, respectively (see Methods).

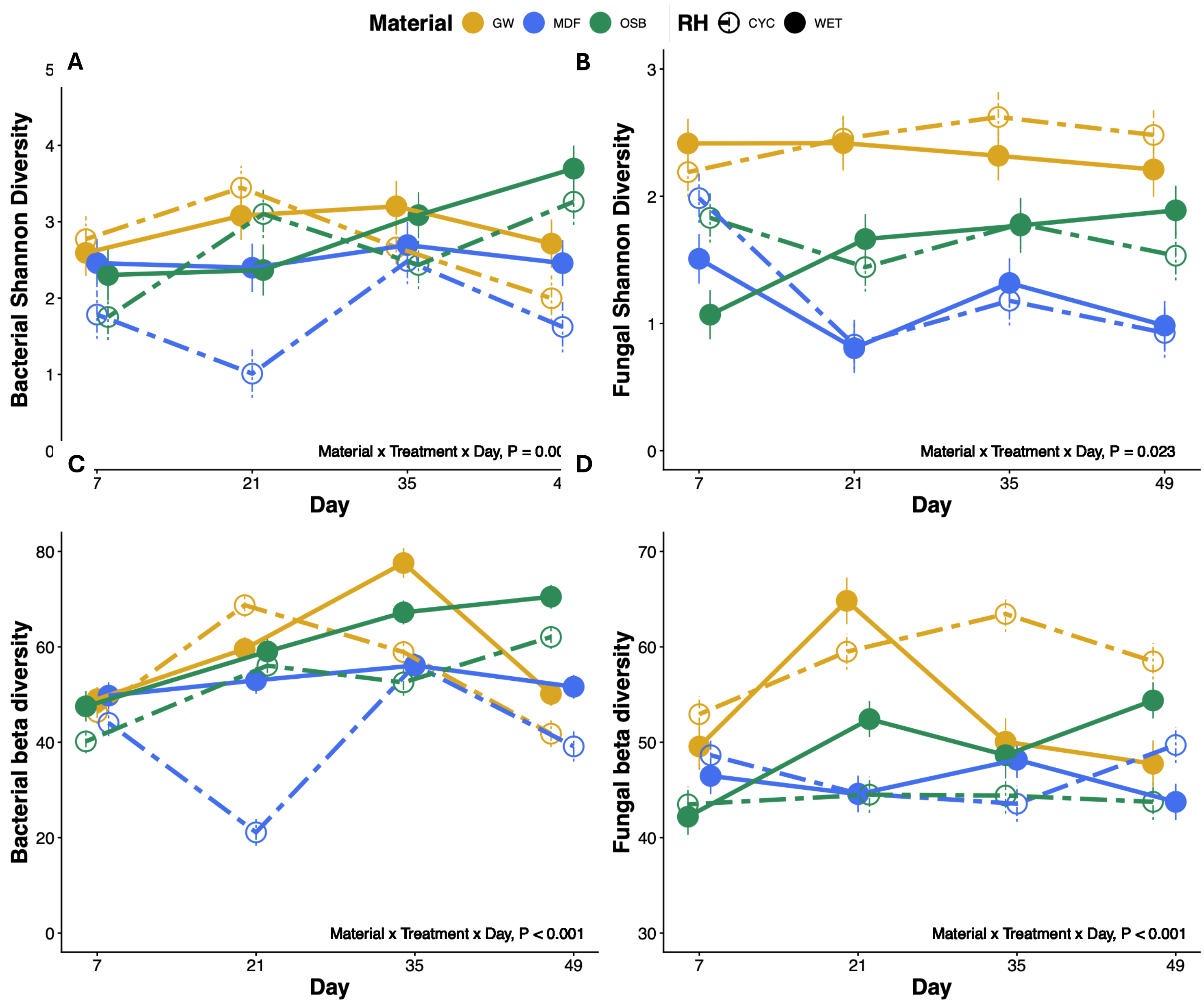

Supplement: Supplementary file 2 [file peerj-14-21859-s002.pdf]
