# Supplementary material for: Moisture and material shape microbial communities in the built environment through disturbance–productivity relationships
Source: PeerJ. 2026 Nov 2;14:e21859. doi: 10.7717/peerj.21859 (PMC13738189; doi:10.7717/peerj.21859)

Figure S3: Comparisons of theoretical to observed patterns of disturbance on productivity. Bacterial (C, D) and fungal (E, F) diversity compared to measures of productivity. Hypothesized relationships from Fig. 1 are reproduced (A, B) for comparison. Values are estimated marginal means ± 1 s.e. Open symbols are the cycling treatment, which corresponds to the orange line in A and B (high disturbance frequently), and closed symbols are the constant wet treatment (blue line, low disturbance frequency).

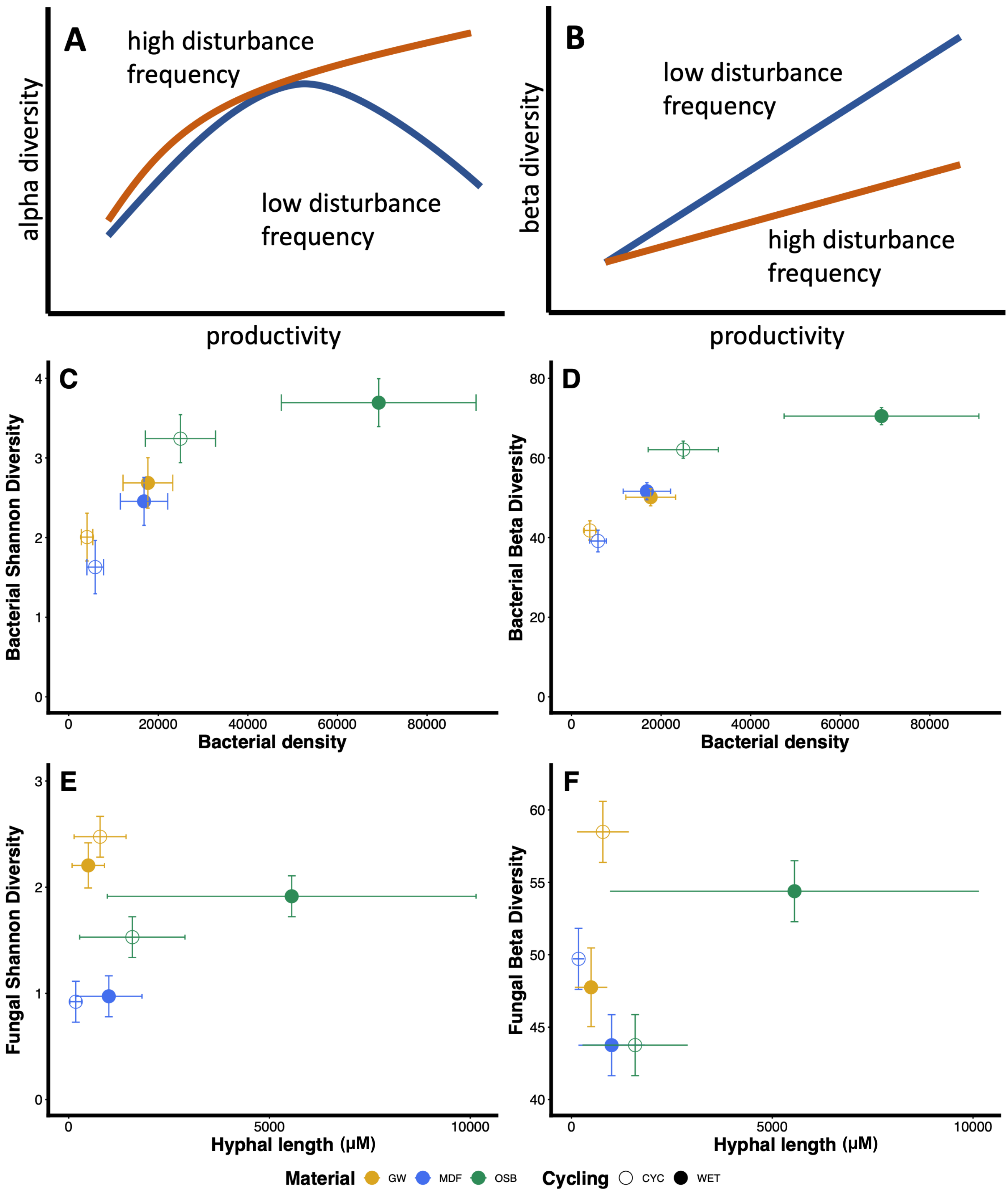

Supplement: Supplementary file 3 [file peerj-14-21859-s003.pdf]
